# Canadian Pandemic Influenza Preparedness: Antiviral strategy

**DOI:** 10.14745/ccdr.v45i01a05

**Published:** 2019-01-03

**Authors:** B Henry

**Affiliations:** 1 Canadian Pandemic Influenza Preparedness Task Group, Chair; 2 Office of the Provincial Health Officer, Victoria, BC

**Keywords:** influenza, pandemic, antivirals, public health

## Abstract

Antiviral medications are the only influenza-specific pharmaceutical intervention that can be used to mitigate the impact of a pandemic until a vaccine becomes available. The *Canadian Pandemic Influenza Preparedness: Planning Guidance for the Health Sector* (CPIP) outlines how federal, provincial and territorial governments will work together to ensure a coordinated and consistent health sector approach to pandemic influenza preparedness and response. This article summarizes Canada’s pandemic influenza antiviral strategy as described in the recently updated CPIP Antiviral Annex. The antiviral strategy builds on lessons learned during the 2009 H1N1 pandemic. Key elements of the strategy include ensuring equitable, timely and coordinated access to antivirals through government stockpiles; having regulatory mechanisms in place that facilitate timely access to antivirals; providing timely and evidence-based clinical guidance; maintaining effective stockpile management practices; and monitoring antiviral utilization, effectiveness and safety. Since the CPIP is an evergreen document, this Annex will be updated as new information warrants.

## Introduction

Antiviral drugs are an integral component of Canada’s pandemic preparedness and response plan. Antivirals are the only influenza-specific pharmaceutical countermeasure that can be used to mitigate the impact of an influenza pandemic in the 4–6 months prior to vaccine availability, using the current egg-based vaccine production technology. The 2009 H1N1 influenza pandemic was the first time the government stockpiles of antivirals were deployed and presented an opportunity to test Canada’s antiviral strategy. The widespread use of antiviral drugs during the pandemic contributed greatly to the evidence base on antiviral safety and effectiveness.

Canada’s renewed antiviral strategy is outlined in the updated *Canadian Pandemic Influenza Preparedness: Planning Guidance for the Health Sector* (CPIP) Antiviral Annex (1,2). The CPIP Antiviral Annex provides technical advice for federal, provincial and territorial (FPT) ministries of health and other government departments that have roles in providing health care to select populations. It describes the specific roles and responsibilities of those involved in providing equitable, coordinated and timely access to antivirals for eligible people living in Canada via FPT stockpiles, enabling regulatory mechanisms, provision of clinical guidance, stockpile management and the monitoring of antiviral use, safety and effectiveness.

The Canadian Pandemic Influenza Preparedness Task Group recently updated the CPIP Antiviral Annex to incorporate the experience gained during the 2009 pandemic and to reflect Canada’s pandemic goals of minimizing serious illness, death and societal disruption. The CPIP Antiviral Annex should be read in conjunction with the main body and other technical annexes of the CPIP as they are intended to be used together.

This article summarizes Canada’s antiviral strategy as outlined in the CPIP Antiviral Annex. It is part of a series outlining Canada’s approach to pandemic influenza preparedness (3–7).

## Objectives of Canada’s Pandemic Antiviral Strategy

The objectives of the antiviral strategy are to support Canada’s pandemic goals by:

- Maintaining and providing timely access to a supply of antivirals
- Reducing the severity and duration of disease through early treatment of influenza cases
- Controlling outbreaks of pandemic influenza in closed health care facilities (e.g., long-term care homes) and other closed facilities (e.g., prisons) and settings (e.g., remote and isolated communities) where residents are at higher risk of severe outcomes of influenza
- Possibly reducing transmission of the influenza virus by reducing the level and duration of viral shedding; and
- Reducing the impact of influenza-related absenteeism in the workplace due to worker illness or family caregiving

### Canadian context

During an influenza pandemic, eligible people will be provided with antivirals by the health authority of the province or territory in which they reside. Some federal departments such as Department of National Defence, Global Affairs Canada, and Correctional Service Canada also have a role in providing and/or administering antivirals to specific populations. Each jurisdiction has its own health care delivery model and will determine how antiviral drugs will be made available during a pandemic.

Canada’s vast and variable geography and diverse population can present challenges to the delivery of antivirals during a public health emergency. Pandemic plans need to take into consideration the impact of adverse weather conditions, long distances from pharmacies and lack of systems that allow timely access to antivirals. As such, pre-positioning of antivirals is an important consideration for provinces and territories with remote and isolated communities.

The diversity of Canada’s population means that jurisdictional pandemic plans need to take into consideration language, culture, ethnicity and religious or spiritual beliefs so that all populations and communities can understand how best to access antiviral treatment in a timely manner.

## Key elements of the antiviral strategy

### Timely access

Timely access to antivirals is fundamental to Canada’s antiviral strategy. Studies conducted during the 2009 influenza pandemic demonstrated that antiviral treatment is most effective when started as early as possible within 48 hours of the onset of symptoms (8,9). The experience identified a number of challenges to providing timely access to antivirals including the triggers for releasing antivirals from stockpiles; logistical issues in distribution; and timeliness of clinical guidance.

There are also unique planning and ethical considerations for individuals whose needs may not be fully addressed by standard services and resources (e.g., those who are culturally or socially isolated, have low income, are recent immigrants). Providing timely treatment requires delivery models that provide rapid access to clinical assessment and make antiviral drugs available through multiple distribution routes (10). The CPIP Antiviral Annex identifies a variety of options for jurisdictions to assess to expedite antiviral treatment including telephone assessment; expanding prescribing authority (e.g., registered nurses or pharmacists); identifying mechanisms for access in communities with limited or no access to health care workers; and providing advance prescriptions.

The CPIP Antiviral Annex demonstrates how other components of Canada’s pandemic preparedness and response are integral to the antiviral strategy including: epidemiological surveillance to inform antiviral decision-making, laboratory surveillance of circulating strains and virus susceptibility to antiviral drugs, health care services to provide access to influenza assessment and antiviral treatment, and timely communications for the public and health care providers on antiviral drug access.

### Antiviral supply

At present, there is no domestic source of influenza antiviral manufacturing in Canada. To ensure timely and equitable access to antiviral drugs across Canada, FPT governments maintain supplies of antivirals, primarily in two stockpiles:

- The National Antiviral Stockpile (NAS), which is the collective name for the antiviral stockpiles held by each province and territory. In the event of a pandemic, eligible people will be provided with antivirals by the province or territory in which they live. The 2009 influenza pandemic marked the first time the provinces and territories released stock from the NAS
- The National Emergency Strategic Stockpile (NESS) is a federal stockpile of emergency pharmaceuticals and medical supplies, including antivirals. The NESS antiviral stockpile is intended to provide surge capacity to provinces/territories if their own NAS supply is depleted. The NESS target size for antiviral holdings is the equivalent of 2.5% population coverage

Following the 2009 influenza pandemic, Canadian experts and decision-makers reviewed the use, size and composition of the NAS. The review included a systematic review of the literature on the safety and effectiveness of neuraminidase inhibitors for seasonal, pandemic and novel influenza; a scan of the antivirals licensed for sale in Canada; an update on the global status of antiviral drug resistance; international stockpiling practices; and mathematical modelling on the optimal size of the NAS. The following is a summary of the final recommendations:

- At the time of an emerging pandemic, the NAS will be used primarily for early treatment, with limited use for post-exposure prophylaxis. Real-time advice will be provided on the use of antivirals, based on available data, to optimize the use of the stockpile
- The size of the NAS should range between 17.14% and 23.19% population coverage. This is based on the projected number of people in Canada that would need treatment in an influenza pandemic with high clinical severity and moderate to high transmissibility. Each province/territory makes its own decision on the size of antiviral stockpile to hold
- The NAS should include oseltamivir (adult and pediatric doses) and other antivirals with different resistance profiles to mitigate the risk of oseltamivir resistance. At this time, the only licensed antiviral meeting this criterion is zanamivir. The recommended proportion of zanamivir is between 18% and 25% of the NAS

These recommendations were endorsed by the Public Health Network Council in 2017 and are expanded upon in the CPIP Antiviral Annex. The NAS and the NESS both currently hold the antivirals oseltamivir and zanamivir, which belong to the neuraminidase inhibitors drug class. FPT governments procure antivirals for their respective pandemic stockpiles through joint supply contracts with antiviral manufacturers. Maintaining stockpiles is a key element of the antiviral strategy because without them there is no guarantee of sufficient supply from commercial markets alone, given that demand for antivirals globally could be high, especially in a more severe pandemic.

### Regulatory mechanisms for emergency access

Health Canada has the authority for licensing drug products, including antiviral drugs, in Canada. In a pandemic, circumstances could arise in which licensed antivirals are not available to adequately treat specific individuals or groups. Such circumstances may include seriously ill patients who are not responding to therapy with licensed antivirals or for certain age groups for which the licensed antivirals are not indicated for use. Health Canada has the legal means to make certain drugs available expediently in a public health emergency. Available mechanisms include an interim order issued by the federal Minister of Health, authorization under Regulations for Extraordinary Use New Drugs, the Special Access Program, a regulatory pathway to enable Access to Drugs in Exceptional Circumstances and authorization of the use of investigational drugs in clinical trials.

### Stockpile management

The 2009 influenza pandemic experience highlighted the need for well-established antiviral stockpile management practices, including storage, distribution and inventory management.

#### Storage

 

In the interpandemic period, the NAS and NESS are held centrally by each FPT jurisdiction in temperature-controlled warehouses or hospital pharmacies.

It is critical that antiviral drugs be stored and transported in a manner that minimizes exposure to conditions (e.g., temperature, humidity and light) that can reduce the drugs’ integrity, quality and efficacy for use during a pandemic. Therefore, antivirals need to be handled according to Good Manufacturing Practices and requirements set out by the manufacturer. The responsibility for maintaining the required storage conditions lies with each party involved in the transportation and storage chain, including dispensing locations.

#### Distribution

 

Effective distribution logistics are crucial to provide timely access to antivirals in a pandemic. Logistics are determined by each province and territory. At the time of a pandemic, jurisdictions will leverage existing distribution systems to distribute the stockpiled antivirals from the central storage facilities to the dispensing locations, while factoring in the environmental impact and geographic conditions. Dispensing locations may include one or more of the following options: community pharmacies; district health authorities; hospitals; community health centres; remote nursing stations; influenza assessment centres; and correctional facilities. To facilitate timely access to antivirals, provinces/territories may also consider delegating prescribing authority to other health care providers, such as registered nurses and pharmacists.

The trigger for releasing antivirals from the NAS for distribution will be based on each jurisdiction’s risk assessment, taking into consideration anticipated timing and impact of the pandemic, as well as the need to ensure access to antivirals from the start of a pandemic virus activity. In some parts of the country, such as in remote and isolated communities, antivirals may need to be pre-positioned (i.e. in advance of pandemic virus activity or even in the interpandemic period) to ensure timely access.

#### Inventory management

 

Real-time inventory management is necessary to track antiviral stockpile capacity and to anticipate shortages. FPT collaboration on real-time data on antiviral holdings and rate of depletion will be important to have by way of a reporting process. Currently, there is no standardized way to collect NAS inventory data. In the 2009 influenza pandemic, each province/territory developed its own method of tracking antiviral distribution and utilization, which included leveraging existing administrative drug formulary systems in retail pharmacies combined with paper-based reporting.

### Clinical recommendations for antiviral use

Guidance for practitioners on the use of antiviral drugs for seasonal influenza is routinely provided by experts from the Association for Medical Microbiology and Infectious Disease Canada (11). Virus-specific guidance for clinicians on the recommended use of antivirals will be provided at the time of an emerging pandemic. The guidance will be based on a risk assessment using the available epidemiology of the pandemic and available scientific evidence, and can be expected to evolve as new information becomes available. Existing scientific expertise will be leveraged to develop clinical guidance and ongoing assessments. The CPIP Antiviral Annex identifies the relevant information to be included in clinical guidance and includes an example of such guidance from the 2009 pandemic.

At the time of a pandemic, guidance may also be provided on the use of antivirals to control outbreaks of pandemic influenza in closed facilities and settings where residents are at higher risk of severe outcomes from influenza. If an antiviral supply shortage is anticipated, antiviral use will be prioritized based on a prioritization framework that is included in the CPIP Antiviral Annex.

### Antiviral safety monitoring

Plans to monitor the safety of antivirals during an influenza pandemic are based on current drug safety monitoring practices. In the event of an influenza pandemic, Health Canada’s Marketed Health Products Directorate will conduct post-market safety surveillance aimed at monitoring, identifying and assessing possible safety issues related to antivirals, developing risk mitigation measures and providing timely communications on potential safety issues identified for these products.

It is expected that reporting adverse reactions will also be based on the current reporting practices. This requires drug manufacturers reporting serious and unexpected adverse reactions that come to their attention to Health Canada. Adverse reaction reports from health professionals and consumers are submitted on a voluntary basis either directly to Health Canada or through the manufacturer.

### Antiviral resistance monitoring

As with seasonal influenza, it is important to have in place a surveillance program to detect antiviral drug resistance during an influenza pandemic. The novel or pandemic influenza virus strain’s susceptibility to antivirals will be tested on an ongoing basis by the National Microbiology Laboratory (NML). Plans call for provincial laboratories to submit a proportion of influenza virus specimens to the NML for antiviral drug resistance testing, as well as testing of samples from clinical situations in which drug resistance is suspected. Information on antiviral susceptibility during the interpandemic period is summarized on a weekly basis in FluWatch reports (12). More information on plans for laboratory testing in a pandemic is available in the CPIP Laboratory Annex (13).

### Risk management approach

Canada’s pandemic antiviral strategy is subject to numerous risks, including the possibility that the pandemic influenza strain is or becomes resistant to the stockpiled antivirals. The CPIP Antiviral Annex incorporates the CPIP new risk management approach to support scalable and flexible pandemic planning, identifying antiviral-specific risks and events and the proposed mitigation. **Table 1** provides an example of how the CPIP risk-based approach is applied to the antiviral strategy.

**Table 1:** Risks affecting the antiviral strategy, their implications and potential mitigation or response

Factor/event	Implications	Potential mitigation/response
Supply of antivirals becoming depleted	Will not be able to treat as many people as anticipated (will impact on pandemic objectives) Health care provider and public distress May not be able to ensure equitable access	Activate measures for surge capacity (e.g., expedited purchases through contracts or advance purchase agreements, NESS, interjurisdictional loans) May need to prioritize antiviral use
Shortage of some specific formulations or products	Unable to provide optimal treatment regimens	Monitor NAS/NESS holdings closely to allow for timely restocking Activate measures for surge capacity, including procurement of needed formulations if available Combine other strengths or compound suspensions to obtain required dose(s) Adjust recommendations and prioritize use
Viral resistance to stockpiled antiviral drugs	Dramatic reduction of available supply of effective antivirals Resistance to all antivirals would effectively remove antiviral treatment option Some groups may be disproportionately impacted, e.g., zanamivir not authorized in young children	Include antivirals with different resistance profiles in NAS Adjust antiviral recommendations and prioritize use Procure effective antivirals, if available If there is resistance to oseltamivir, consider authorizing lower age for zanamivir diskhaler use Engage rapid clinical research into effective regimens

Abbreviations: NAS, National Antiviral Stockpile; NESS, National Emergency Strategic Stockpile

## Discussion

Canada’s pandemic influenza preparedness and response require a multifaceted approach. Antiviral drugs are an essential component, being the only pharmaceutical intervention until vaccine becomes available.

Central to Canada’s pandemic antiviral strategy is the ability to provide timely access to a secure government controlled supply of safe and effective antivirals. Since the 2009 influenza pandemic, the antiviral strategy has been strengthened through updated recommendations for the NAS; identification of best practices in stockpile management; new regulatory pathways to make certain drugs available expediently; strategies to provide timely clinical guidance in a pandemic; and plans for safety surveillance for antiviral drugs in a pandemic. In addition, antiviral drug susceptibility is monitored on an ongoing basis by the NML. The risks to the antiviral strategy have been identified and mitigation strategies proposed for jurisdictions to consider in their pandemic planning.

Canada’s antiviral strategy is subject to change based on advances in antiviral drug research and development. Since the CPIP is an evergreen document, the Antiviral Annex will be updated as required.

The Antiviral Annex also demonstrates the importance and linkage of other pandemic preparedness components to the antiviral strategy including surveillance, laboratory monitoring, access to health care services, and timely communication strategies. The Annex outlines how all levels of government have a crucial role in ensuring timely and equitable access to antivirals for Canadians at the time of an influenza pandemic.
